# A Long Noncoding RNA, GAS5 Can Be a Biomarker for Docetaxel Response in Castration Resistant Prostate Cancer

**DOI:** 10.3389/fonc.2021.675215

**Published:** 2021-05-21

**Authors:** Yuting Shan, Yingbo Huang, Adam M. Lee, Joshua Mentzer, Alexander Ling, R. Stephanie Huang

**Affiliations:** Department of Experimental and Clinical Pharmacology, University of Minnesota, Minneapolis, MN, United States

**Keywords:** long noncoding (lnc) RNA, GAS5, castration-resistant prostate cancer (CRPC), docetaxel, drug resistance, *ABCB1*

## Abstract

While functional studies of long noncoding RNAs (lncRNAs) have mostly focused on how they influence disease diagnosis and prognosis, the pharmacogenomic relevance of lncRNAs remains largely unknown. Here, we test the hypothesis that the expression of a lncRNA, grow arrest-specific 5 (*GAS5*) can be a biomarker for docetaxel response in castration resistant prostate cancer (CRPC) using both prostate cancer (PCa) cell lines and CRPC patient datasets. Our results suggest that lower *GAS5* expression is associated with docetaxel resistance in both PCa cell lines and CRPC patients. Further experiments also suggest that *GAS5* is downregulated in docetaxel resistant CRPC cell lines, which reinforces its potential as a biomarker for docetaxel response. To examine the underlying biological mechanisms, we transiently knockdown *GAS5* expression in PCa cell lines and then subject the cells to docetaxel treatment overtime. We did not observe a decrease in docetaxel induced growth inhibition or apoptosis in the siRNA treated cells. The findings suggest that there is no direct causal relationship between change in *GAS5* expression and docetaxel response. Subsequently, we explored the indirect regulation among *GAS5*, ATP binding cassette subfamily B member 1 (*ABCB1*), and docetaxel sensitivity. We showed that transient knockdown *GAS5* did not lead to significant changes in *ABCB1* expression. Therefore, we rule out the hypothesis that *GAS5* directly down regulate *ABCB1* that lead to docetaxel sensitivity. In conclusion, our work suggests that *GAS5* can serve as a predictive biomarker for docetaxel response in CRPC; however, the exact mechanism behind the observed correlation remain to be elucidated.

## Introduction

Long noncoding RNAs (lncRNAs) are defined as transcripts greater than 200 nucleotides with no protein-coding capacities. Constituting the majority of the non-coding transcriptome (1), lncRNAs are shown to be involved in essential biological processes at both transcriptional and posttranscriptional levels including but not limited to chromosome silencing, RNA processing, and protein-RNA interactions (2, 3). Most recently, emerging evidence has shown that certain lncRNAs play important roles in human cancers by functioning as either oncogenes or tumor suppressor genes (4–6).

The growth arrest-specific arrest 5 (*GAS5*) lncRNA was originally identified by Schneider et al. as being preferentially expressed in grow-arrested cells (7). Later studies have suggested *GAS5* as a tumor suppressor gene in various types of cancer through inhibiting proliferation, invasion and promoting apoptosis (8–11). In addition to its potential suppressor role in tumor growth, *GAS5* has also been shown to be associated with the response of several anticancer agents such as docetaxel, doxorubicin, and tamoxifen (12–14). A recent lncRNA study that systematically surveyed the pharmacologic role of lncRNAs shows that the expression of *GAS5* correlates with the sensitivity of over 100 anti-cancer drugs in a collection of pan-cancer cell lines (15). This work suggests that *GAS5* may be a master biomarker for the response to chemotherapeutics in various cancer types.

Prostate cancer (PCa) is the most commonly diagnosed cancer in men, and it consists of about 8% of the total cancer deaths in male population in the US (16). 10- 50% of all PCa patients will advance to castration resistant prostate cancer (CRPC) within three years of diagnosis and CRPC accounts for nearly all PCa mortality (17). For CRPC patients, docetaxel is recommended clinically as the first-line treatment (18). Unfortunately, patients’ response to docetaxel varies and there is no clinically actionable biomarker to predict its efficacy in CRPC patients (19).

In this study, we set out to examine the value of *GAS5* expression in predicting docetaxel response in CRPC. Furthermore, after confirming the correlative nature of *GAS5* expression and docetaxel response, we explore the potential underlying biology based on a hypothesis that *GAS5* achieves its docetaxel sensitizing role by affecting ABCB1, whose expression level was shown to be upregulated in CRPC in previous studies (20, 21).

## Materials and Methods

### Data Acquisition

The transcriptome profiles (in the form of RPKM) of cancer cell lines were obtained from the Cancer Cell Line Encyclopedia (CCLE, https://portals.broadinstitute.org/ccle/data, release date 01/02/2019). *In vitro* drug response data were obtained from the Cancer Therapeutics Response Portal (CTRPv2, https://ocg.cancer.gov/programs/ctd2/data-portal/, CTRPv2.1_2016). Transcriptome profiles and *in vitro* drug response data were also obtained from an independent high throughput cancer cell line drug screening dataset: Genomics of Drug Sensitivity in Cancer (GDSC) (https://www.cancerrxgene.org/) as the training dataset for docetaxel sensitivity imputations in PCa patients. In addition, a total of 3 PCa clinical studies were evaluated including 1) The Cancer Genome Atlas (TCGA) prostate cancer, with RNA-seq data (in the form of FPKM) and clinical phenotypes obtained from the University of California, Santa Cruz Xena browser (https://xenabrowser.net/datapages/, Release 18.0); 2) The Prostate Cancer Medically Optimized Genome-Enhanced Therapy (PROMOTE) trial, with RNA-seq data (in the form of RPKM) clinical phenotypes obtained from dbGaP (https://www.ncbi.nlm.nih.gov/gap/), study ID: phs001141.v1.p1; 3) Stand Up To Cancer (SU2C) prostate cancer study, with RNA-seq data (in the form of RPKM) and clinical phenotypes obtained from cBioPortal (https://www.cbioportal.org/). Gene expression data were normalized using the log_2_(FPKM + 1) or log_2_(RPKM + 1) method.

### Treatment Response of Docetaxel in PCa Cell Lines and PCa Patients

To evaluate the relationship between *GAS5* expression and docetaxel sensitivity, we first obtained *GAS5* expression from RNAseq data in CCLE, PROMOTE, and SU2C. The docetaxel response data were generated from both *in vitro* and *in vivo* sources. For *in vitro* assessment, the area under the docetaxel dose-response curve (AUC) parameter from all cancer cell lines was obtained from CTRPv2. To assess the docetaxel response in PCa patients, we imputed docetaxel drug sensitivity score (DSS) using *“pRRophetic”* package, a computational tool that has been shown to accurately impute patient tumor response to various anti-cancer agents (22). More specifically, in this study, we built the relationship matrix using the transcriptome and measured docetaxel response data from GDSC before imputing docetaxel sensitivity score in PCa patients. We used GDSC instead of CPRPv2 as the training dataset is for the reason that the docetaxel concentration range screened in GDSC is more clinically relevant than that in CTRPv2. Pearson’s correlation coefficient was calculated between the *GAS5* expression and docetaxel response as represented by either measured docetaxel AUC in cancer cell lines or docetaxel DSS in PCa patients.

### Expression Correlation Between *GAS5* and *ABCB1*

Again, the expression levels of *GAS5* and *ABCB1* were obtained from both cancer cell lines (CCLE) and PCa patients (TCGA-PRAD, PROMOTE, SU2C) RNA-seq data. Pearson’s correlation coefficient was calculated between the expression of *GAS5* and *ABCB1* in cancer cell lines and PCa patients.

### Cell Culture and Reagents

We obtained the human PCa cell line DU145 from ATCC (https://www.atcc.org/) and another PCa cell line, R1D567 from the Dehm laboratory (23). Both cell lines were cultured in RPMI 1640 medium (Thermo Fisher Scientific) supplemented with 10% fetal bovine serum (FBS) (Gibco, Thermo Fisher Scientific). Cells were maintained in a humidified atmosphere of 5% CO_2_ at 37°C and were observed periodically to confirm morphology.

### Docetaxel Resistant Cell Lines Development

Docetaxel resistant prostate cancer cell lines were established over 3 months by chronically exposing the parent cell lines to stepwise increasing concentrations of docetaxel. In brief, the cell lines were initially exposed to docetaxel at a concentration of 2×IC_50_ of the respective parent cell lines for 72 hours. Then, they were cultured under the regular medium and continuously monitored under the microscope until colonies formed. The cell lines were resuspended into a new T25 flask and the treatment cycle was repeated with the same concentration of docetaxel when the cell lines reached 50% confluency. As cells displayed resistance to docetaxel, the concentration was subsequently increased to 3×IC_50_, 4×IC_50_, and the treatment cycle was repeated as described above. The resistance was determined by the decrease of cell death during exposure to docetaxel. Control cells were maintained under the same percentage of the vehicle (DMSO) as resistant cell lines. All cell lines were within a passage number of 20.

### Knock Down *GAS5* through siRNA

R1D567 cells were plated in 6-well plates under a seeding density of 2 × 10^5^ cells/well. Cells were reversely transfected with Lincode *GAS5* Control Pool (Human) using DharmaFECT reagent 2 according to the manufacturer-supplied protocol. Transfection reagent was removed after 48 hours due to potential cytotoxicity. The final siRNA concentration was 25 nM and the final concentration of DharmaFECT reagent 2 was 0.1%. Four different siRNAs were employed for *GAS5* knockdown (catalog ID: D-001310-1): GAUGGAGUCUCAUGGCACA, UGGAUGACUUGCUUGGGUA, AGGUAUGGAGAGUCGGCUU, AGGCAGACCUGUUAUCCUA. Scramble control was transfected with negative control siRNA (catalog ID: D-001320-10): UGGUUUACAUGUCGACUAA, UGGUUUACAUGUUGUGUGA, UGGUUUACAUGUUUUCUGA,UGGUUUACAUGUUUUCCUA.

### Real-Time Reverse Transcriptase PCR

We obtained total RNA from cultured cells using the Quick-RNA MiniPrep Plus Kit (Zymo Research, Irvine, CA). The NanoDrop ND-8000 spectrophotometer (Thermo Fisher Scientific, Waltham, MA) was used for RNA quantification. cDNA was synthase using The High Capacity cDNA Reverse Transcription Kit (Thermo Fisher Scientific, Waltham, MA). The Sso-Advanced Universal SYBR Green SuperMix (Bio-Rad, Hercules, CA) and the 7500 Real-Time PCR System (Applied Biosystems, Foster City, CA) were used to conduct real-time PCR analyses under manufacturer’s protocol. The PCR primers used to amplify target gene/lncRNA and housekeeping genes are as follows: *ABCB1* Forward 5’ – GATGCTGGTGTTTGGAGAAATG, *ABCB1* Reverse 5’ - GCCTATCTCCTGTCGCATTATAG, *GAS5* Forward 5’ – TGGATGACTTGCTTGGGTAAG, *GAS5* Reverse 5’ – TAACAGGTCTGCCTGCATTT, *GAPDH* Forward 5’- GAACATCATCCCTGCCTCTAC -3’, *GAPDH* Reverse 5’- CCTGCTTCACCACCTTCTT -3’. All results were normalized with the expression of *GAPDH*. Expression results were quantified using the ΔΔCt method relative to the scramble control.

### Determine Docetaxel Response Through Growth Inhibition and Apoptosis Assay After *GAS5* Knockdown

R1D567 cells were trypsinized, harvested, counterstained with Hoechst 33342 Fluorescent Stain (Thermo Scientific, Pierce Biotechnology, Rockford, IL) and resuspended in full growth media to 1x10^5^ cells per mL prior to plating and reverse transfection with siRNAs as previously described. Cells were plated in 96-well microplates (Thermo Scientific) using a seeding density of 1x10^4^ cells per well and allowed to attach and transfect with appropriate siRNA for 48 hours. Following incubation, cells were treated with different concentrations of docetaxel ranging from 1nM to 16nM. Apoptosis was kinetically measured over 72 hours using the CellEvent™ Caspase-3/7 Green Detection Reagent (Invitrogen, Life Technologies, Carlsbad, CA) following manufacturer’s protocol. Cell culture plates were transferred by the BioSpa 8 to a Cytation™ 1 Cell Imaging Multi-Mode Reader (BioTek Instruments, Winooski, VT) every six hours, and images were captured in the DAPI, GFP, and brightfield channels. Automatic background flattening parameters were used to remove background fluorescence from the GFP and DAPI channels. Object masking thresholds were then set to identify each cell for counting. Total cell counts per well were obtained using the DAPI fluorescence intensity > 2000 as threshold. Apoptotic cell count was obtained as a subpopulation of the total cell count using the GFP fluorescence intensity > 2000 as threshold. Results are reported as a mean and standard deviation of two independent biological experiments, each containing three technical replicates for each experimental condition.

Cell viability after docetaxel treatment was also measured after knocking down *GAS5* with siRNAs as previously described. R1D567 (5x10^3^ cells per well) and DU145 cells (3x10^3^ cells per well) were plated in 96-well microplates (Thermo Scientific) and allowed to attach and transfect with appropriate siRNA for 24 hours. Cells were then exposed to various concentrations of docetaxel for 72 hours. Cell viability was measured using WST-1 assay [(Roche Applied. Science, Penzberg, Upper Bavaria, Germany) following the manufacturer’s protocol. Absorbance at the 450nm wavelength was assessed using the Synergy HTX Multi-Mode Plate Reader (BioTek, Winooski, VT)].

### Statistical Analysis and Software

All imputations, predictions, and statistical analyses were performed in the R statistical computing environment. The drug sensitivity curves were plotted and analyzed to obtain the IC_50_ values using GraphPad Prism 7.0. A p value of < 0.05 was considered statistically significant.

## Results

### *GAS5* as a Biomarker for Docetaxel Sensitivity in CRPC

**Figure 1:**
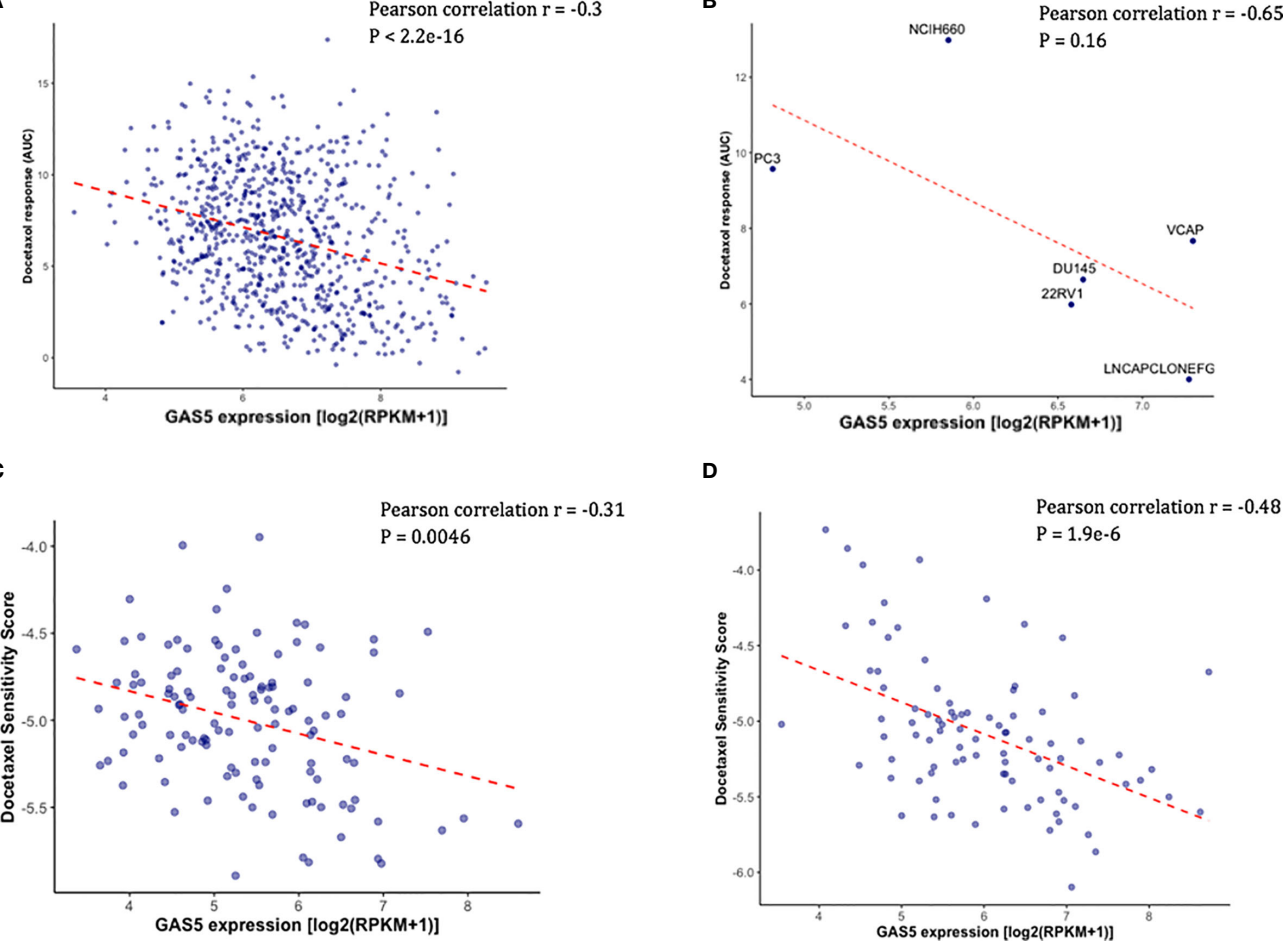
Higher *GAS5* expression was associated with higher docetaxel sensitivity in cancer cell lines and CRPC patients. Lower imputed docetaxel sensitivity score indicates higher sensitivity in CRPC patients. **(A)** Scatter plot showing docetaxel response (AUC) (Y-axis) and *GAS5* expression in 811 cancer cell lines in CTRPv2. **(B)** Scatter plot showing docetaxel response (AUC) (Y-axis) and *GAS5* expression in 6 PCa cell lines. Name of the cell line was identified above the corresponding point. **(C)** Scatter plot showing imputed docetaxel sensitivity score (Y-axis) and measured *GAS5* expression in 117 CRPC patients in SU2C clinical study. **(D)** Scatter plot showing imputed docetaxel sensitivity score (Y-axis) and measured GAS5 expression in 91 CRPC patients in PROMOTE trial.

Using the existing *in vitro* docetaxel sensitivity screening dataset (CTRPv2) and cancer cell line RNA-seq dataset (CCLE), we first assessed the correlation between the expression of *GAS5* and the measured docetaxel sensitivity across 811 cancer cell lines. As expected, a significant negative correlation of *GAS5* expression and the AUC of docetaxel has been observed, with higher docetaxel sensitivity in higher *GAS5* expressed cells (Pearson correlation coefficient r = -0.3, P < 2.2 x 10^-16^) (**Figure 1A**). The same directional effect was also observed in a small collection of 6 PCa cell lines available in CTRPv2 (Pearson correlation coefficient r = -0.65, P = 0.16) (**Figure 1B**). To evaluate the *GAS5* and docetaxel response relationship in CRPC patients, we obtained the *GAS5* expression from two independent CRPC patient datasets: SU2C and PROMOTE. In both studies, higher expression levels of *GAS5* correlated with higher docetaxel sensitivity (as indicated with higher imputed docetaxel response) in prostate cancer patients (SU2C: Pearson correlation coefficient r = -0.31, P = 0.0046, PROMOTE: Pearson correlation coefficient r = -0.48, P < 1.9 x 10^-6^ (**Figures 1C**, **D**). These results from both cancer cell lines and PCa patients suggest the potential of *GAS5* expression as a predictive biomarker for docetaxel sensitivity in prostate cancer.

### Docetaxel Resistant Prostate Cancer Cell Lines Exhibit Decreased *GAS5* Expression

**Figure 2:**
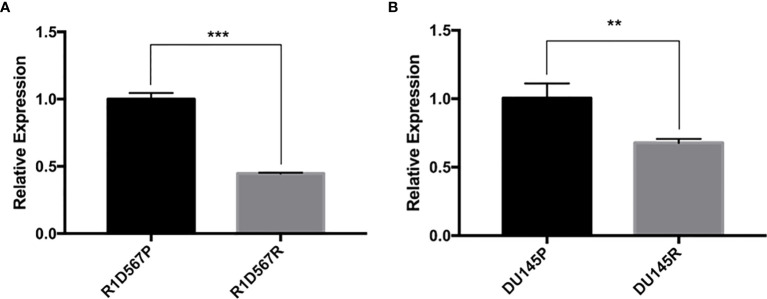
Docetaxel resistant PCa cell lines exhibit lower *GAS5* expression when compared to parent docetaxel sensitive PCa cell lines. The expression was measured using real-time RT-PCR and was normalized to GAPDH expression. **(A)** Comparison of relative *GAS5* expression levels in R1D567 parental line (R1D567P, black bar) and docetaxel resistant line (R1D567R, grey bar). **(B)** Comparison of relative *GAS5* expression levels in DU145 parental line (DU145P, black bar) and docetaxel resistant line (DU145R, grey bar). **P < 0.01, ***P < 0.001.

Two PCa cell lines (DU145 and R1D567) were used as the CRPC models for the experimental validation. DU145 is widely used as a CRPC cell line model as it is insensitive to anti-hormonal treatment. R1D567 is a genetically engineered cell line derived from a R1AD1 prostate cancer cell line, which was isolated from a CRPC patient. The exons 5-7 were deleted from the androgen receptor gene in R1AD1 using TALEN to establish R1D567. As a result, R1D567 cells are androgen independence (23). We established docetaxel resistant cell lines (DU145R and R1D567R) by chronically exposing parent cell lines to increasing concentrations of docetaxel. The resulting resistant cell lines have the following features: DU145R tolerates 2 × IC_50_ (2 x 6 nM) docetaxel treatment and R1D567R tolerates 4 × IC_50_ (4 x 3 nM) docetaxel. *GAS5* expression was measured in both docetaxel resistant cell lines and their corresponding DMSO treated control cell lines. In DU145R, *GAS5* was downregulated by nearly 40% compared to the DU145 control cell line (P = 0.0068) (**Figure 2A**). Similarly, compared to the parent R1D567 DMSO treated cell line, *GAS5* was significantly downregulated by over 50% in R1D567R (P = 0.0001) (**Figure 2B**). These results provided additional support for the role of *GAS5* in docetaxel sensitivity in CRPC.

### Transient *GAS5* Knockdown Did Not Decrease Percentage Apoptosis or Increase Percentage of Viable Cells After Docetaxel Treatment

To evaluate whether there is a causal relationship between *GAS5* expression level and docetaxel sensitivity in CRPC, we transiently knocked down *GAS5* using siRNA pool in R1D567 before treating the cells with different concentrations of docetaxel. According to qPCR results, *GAS5* knockdown efficacy was greater than 75% at 48h, 72h, and 96h (**Supplementary Figure 1**). Then, we assessed cell proliferation in both *GAS5* knockdown and controlled R1D567 cells. If lower *GAS5* expression led to docetaxel resistance, we would observe a higher proliferation rate in *GAS5* knockdown group. However, our results showed that there was no difference in cell proliferation between the two groups (**Supplementary Figure 2**). Since docetaxel exerts its cytotoxic effect mainly through inducing cell apoptosis, we then investigated whether knocking down *GAS5* would change percentage apoptosis under docetaxel treatment. We did not observe a decrease in percentage apoptosis under docetaxel treatment after knocking down *GAS5* (**Supplementary Figure 3**). In fact, we found a slight but significant increase in percentage apoptosis in the siRNA treated group after exposing to 2-4nM of docetaxel. The cell proliferation and apoptosis results together suggest that there is no short-term causal relationship between *GAS5* expression and docetaxel sensitivity.

### *GAS5* Expression Negatively Correlated With *ABCB1* Expression in PCa Patients

**Figure 3:**
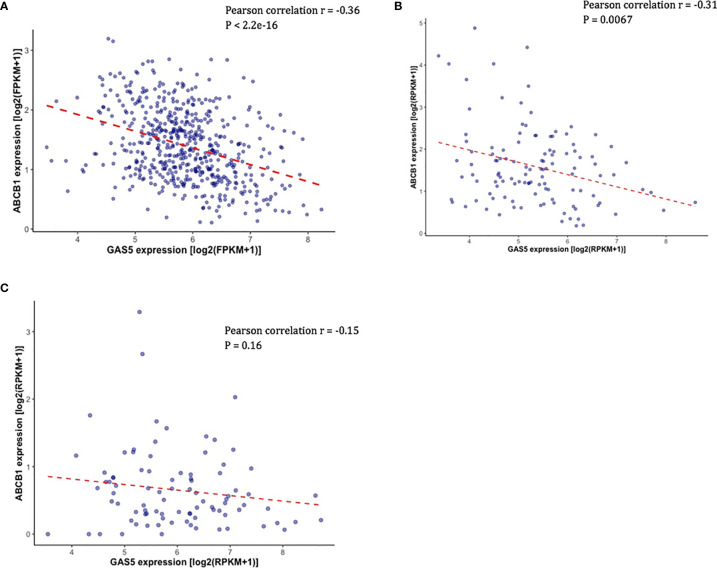
Validation of the correlation between the expression of *GAS5* and the expression of *ABCB1* in PCa patient datasets. **(A)** Scatter plot showing the expression level of *ABCB1* (Y-axis) and the expression level of *GAS5* in PCa patients in TCGA. **(B)** Scatter plot showing the expression level of *ABCB1* (Y-axis) and the expression level of *GAS5* in PCa patients in SU2C. **(C)** Scatter plot showing the expression level of *ABCB1* (Y-axis) and the expression level of *GAS5* in PCa patients in PRMOTE.

Overexpression of *ABCB1* is known to be associated with docetaxel resistance in CRPC cell lines, and knockdown of *ABCB1* has been shown to be able to re-sensitized resistant cell lines to docetaxel (20). Given that *ABCB1* expression is functionally associated with docetaxel resistance in CRPC, we sought to determine if there is a link between the expression of *GAS5* and *ABCB1*. In TCGA-PRAD, the expression of *ABCB1* was found to be negatively correlated with *GAS5* expression (Pearson correlation coefficient r = -0.36, P < 2.2 x 10^-16^) (**Figure 3A**). In addition, the same directional correlations were observed in CRPC patients in SU2C and PROMOTE datasets (SU2C Pearson correlation coefficient r = -0.31, P = 0.0067, PROMOTE Pearson correlation coefficient r = -0.15, P = 0.16) (**Figures 3B**, **C**).

### *ABCB1* Was Found to Be Upregulated in Both Docetaxel Resistant PCa Cell Lines and Predicted Poor Docetaxel Responders

**Figure 4:**
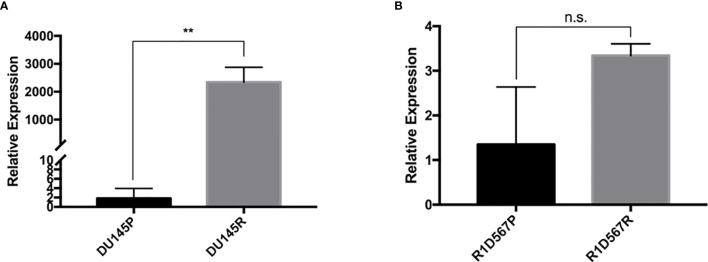
In vitro validation of *ABCB1* dysregulation in docetaxel response in CRPC cell lines. The expression was measured using real-time RT-PCR and was normalized to GAPDH expression. **(A)** Comparison of relative *ABCB1* expression levels in DU145 parental line (black bar) and docetaxel resistant line (grey bar). **(B)** Comparison of relative *ABCB1* expression levels in R1D567 parental line (black bar) and docetaxel resistant line (grey bar). **P < 0.01, n.s., not significant.

**Figure 5:**
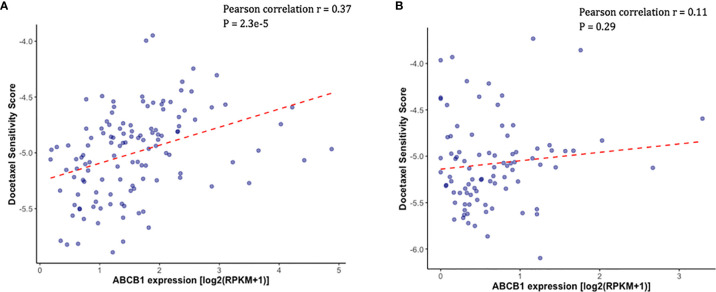
Higher measured *ABCB1* expression was correlated with higher predicted docetaxel sensitivity score (indicating lower sensitivity to docetaxel) in two CRPC trials. **(A)** Scatter plot showing the predicted docetaxel sensitivity score (Y-axis) and the expression level of *ABCB1* (X-axis) in PCa patients in SU2C trial. **(B)** Scatter plot showing the predicted docetaxel sensitivity score (Y-axis) and the expression level of *ABCB1* (X-axis) in PCa patients in PROMOTE trial.

Docetaxel is a well-established substrate of *ABCB1*. We measured *ABCB1* expression level in both docetaxel resistant and control cell lines. In DU145R, *ABCB1* was significantly upregulated compared to the corresponding DMSO treated control cell line (P = 0.0031) (**Figure 4A**). Similarly, although not statistically significant, upregulation of *ABCB1* was also observed in R1D567R compared to the control (P = 0.1) (**Figure 4B**). Furthermore, we observed a negative correlation between *ABCB1* expression and imputed docetaxel sensitivity in two CRPC trials. In SU2C, higher *ABCB1* expression was found to be correlated with lower predicted docetaxel sensitivity (Pearson correlation coefficient r = 0.37, P = 2.3 x 10^-5^) (**Figure 5A**). Similar direction was also found in PROMOTE trial (Pearson correlation coefficient r = 0.11, P = 0.29) (**Figure 5B**).

### Transient *GAS5* Knockdown Did Not Change *ABCB1* Expression in CRPC Cell Lines

Since we did not observe a direct effect of *GAS5* knockdown to docetaxel response, we hypothesized that GAS5 may indirectly affect docetaxel response through its regulation on *ABCB1* gene. We performed *GAS5* knockdown experiments in R1D567 using a pooled siRNA targeting *GAS5*. The expression level of *ABCB1* was measured 48h, 72h, and 96h after *GAS5* knockdown. We expected that knocking down *GAS5* would result in *ABCB1* upregulation. However, qPCR results showed that the expression level of *ABCB1* was not significantly changed after *GAS5* being knocked down (**Supplementary Figure 4**).

## Discussion

Despite the significant advances in cancer treatment, CRPC remains a deadly disease. Docetaxel is a first-line therapy for CRPC. However, the response to this key therapy varies largely between individuals (24). Currently, with additional treatment options becoming available for CRPC patients such as radium 223, sipuleucel, and cabazitaxel, there is an urgent need to identify actionable biomarkers to predict docetaxel sensitivity in CRPC so the patients can be triaged to different therapies if he is not going to respond to docetaxel therapy. In this study, we leveraged existing *in vitro* and *in vivo* data for hypothesis generation and performed experimental validation to identify the role of *GAS5*, a *lncRNA*, as a predictive biomarker for docetaxel response in CRPC.

As a well-established tumor suppressor gene, *GAS5* is shown to exert growth arrest effects in different types of cancer. Luo et al. demonstrated that *GAS5* enhances the promoter activity of cyclin-dependent kinase inhibitor 1B (P27Kip1), a regulator of cell cycle, by enhancing the binding of E2F1 to P27Kip1 promotor in prostate cancer (10). Moreover, *GAS5* was shown to inhibit pancreatic cancer cell proliferation by inhibiting the transcriptional activity of glucocorticoid receptor (GR) in CD133+ population which is often attributed to tumor metastasis and recurrence (25). Besides acting as a tumor suppressor, *GAS5* was also found to confer pharmacogenomic significance in various types of cancer. Yacqub-Usman et al. showed that in androgen-dependent prostate cancer, mTOR inhibitors induced *GAS5* upregulation and knocking down *GAS5* resulted in resistance to mTOR inhibitors (26). Similarly, Li et al. demonstrated that *GAS5* downregulation led to trastuzumab resistance in breast cancer, and the resistance could be alleviated by lapatinib which inhibits PI3K/Akt/mTOR pathway and eventually upregulates *GAS5* (27). In this study, we focused on the role of *GAS5* in docetaxel response in CRPC. The initial analysis in CTRP dataset suggested that more *GAS5* expression was associated with high docetaxel sensitivity in cancer cell lines. Then, after establishing docetaxel resistant cell line models by chronically exposing two CRPC cell lines (DU145 and R1D567) to docetaxel, we found that *GAS5* was downregulated by nearly 50% in both docetaxel resistant cell line models. These findings support the predictive value of *GAS5* as a docetaxel response biomarker in CRPC.

Beyond the *in vitro* findings, we also investigated whether the association between *GAS5* expression and docetaxel sensitivity can be recapitulated in CRPC patients. By utilizing both RNAseq data and predicted docetaxel sensitivity score, we further demonstrated that higher *GAS5* expression correlated with higher predicted docetaxel sensitivity in CRPC patients through analysis of two independent clinical studies (PROMOTE and SU2C). Notably, even if some of the patients in PROMOTE and SU2C received docetaxel as one of their treatments, the RNAseq was obtained before they physically got the drug. Therefore, the predictive role of *GAS5* becomes more valuable and clinically actionable since the docetaxel sensitivity can be predicted before the treatment starts.

To test the hypothesis that *GAS5* expression is a direct cause of docetaxel response in CRPC, we performed *GAS5* knockdown in R1D567 and then the cellular sensitivity to docetaxel (measured through cellular growth inhibition and cell apoptosis after docetaxel exposure) were compared between the *GAS5* knockdown and control groups. We found that transient knockdown of *GAS5* did not result in docetaxel resistance within 72 hours. This finding is in conflict with a previously reported *in vitro* study, in which short-term knockdown of *GAS5* diminished the cell-killing effects of docetaxel in prostate cancer cells (12). Further examination revealed that the cell line model utilized in the previous study was 22Rv1, a hormone sensitive PCa cell line (28). Therefore, the discrepancy in the observation may be in part due to the model system employed. Our results suggest that there is no direct causal relationship between *GAS5* expression and docetaxel response in CRPC cell lines.

We then hypothesized that *GAS5* can indirectly affect docetaxel resistance by influencing other coding genes and subsequently lead to docetaxel resistance. To test this hypothesis, we first chose to examine a potential intermediate gene, *ABCB1*. This gene encodes a major transporter, P-glycoprotein (P-gp), that pumps out foreign substances in an ATP-dependent manner. Our rationale to focus on this gene is that *ABCB1* has been known to be related to chemoresistance in many different types of cancer (29–31). Recent studies have shown that *ABCB1* could potentially modulate docetaxel resistance in prostate cancer (20, 21). Indeed, we confirmed these literature findings upon examining the correlation between *ABCB1* gene expression and imputed docetaxel response scores in both clinical studies. In addition, we found that the expression of *ABCB1* was increased in both DU145R and R1D567R docetaxel resistant cell lines. We also observed a negative correlation between the expression of *GAS5* and *ABCB1* in CRPC patients in both PROMOTE and SU2C trials. These findings suggest that there may be a link either directly or indirectly between *GAS5* and ABCB1.

To investigate whether there is a direct interaction between *GAS5* and ABCB1, we performed *GAS5* knockdown experiments in R1D567 to explore whether transient knockdown of *GAS5* would lead to *ABCB1* overexpression. To this end, we observed no *ABCB1* expression change at 48h, 72h, and 96h after *GAS5* knockdown. These results are in contrast to our observations in docetaxel resistant cell models, which were established through chronic docetaxel exposure (months as compared to the siRNA experiments which only go up to 96 h). In the docetaxel resistant cell lines, we found significant decrease in *GAS5* expression and significant increase in *ABCB1* expression. Taken together, these results suggest that the expression of *GAS5* did not directly affect *ABCB1* expression. Therefore, we speculate that there is an indirect relationship between GAS5 and ABCB1; and that it will take time for the impact of *GAS5* expression to show both at the *ABCB1* expression level and for docetaxel response. This theory has support from a study by Xiao Lin et al, in which the authors suggest that the expression and function of lncRNAs may be time-dependent (32).

There are several limitations to our study. First of all, instead of the measured docetaxel response in prostate cancer patients, we utilized the docetaxel sensitivity score imputed using patients’ RNAseq data to perform the correlation between *GAS5* expression and docetaxel response. The reasons behind this decision are 1. The lack of recorded docetaxel response in CRPC patients with measured tumor RNA data; 2. multiple treatment modalities leads to difficulty in attributing survival outcome to any specific treatment agents. It is worth noting that the drug sensitivity imputation model employed in this study has been proven to be able to capture a large proportion of response variability in different clinical trials (22). A second limitation of our study is that, utilizing transient *GAS5* knockdown by siRNA, we were unable to track the long-term effects of *GAS5* dysregulation in CRPC cell lines. Even though we observe dysregulation of both *GAS5* and *ABCB1* in DU145R and R1D567R and their correlation in PCa patients, our experimental findings suggest that there is no direct regulatory relationship between *GAS5* expression and docetaxel sensitivity nor with *ABCB1* expression. Therefore, the exact mechanism behind the *GAS5* expression and docetaxel sensitivity correlation is yet to be elucidated.

In summary, we have identified that a lncRNA, *GAS5*, can potentially serve as a predictive biomarker for docetaxel sensitivity in CRPC. Our findings were validated in both PCa cell lines and PCa patient data. Given the urgent clinical need for biomarkers to predict docetaxel sensitivity in CRPC, we expect future work in this area to compare performance of various new biomarkers, including ours and to explore the underlying biological mechanisms to fully appreciate *GAS5* as a biomarker for docetaxel response and promote its clinical application.

## Data Availability Statement

The original contributions presented in the study are included in the *Date Acquisition* section under *Materials and Methods*. Further inquiries can be directed to the corresponding author.

## Author Contributions

RH and YS conceived the idea. YS, AML, and YH designed and performed the experiments. JM and AL imputed docetaxel sensitivity scores in PCa patients. YS wrote the manuscript. All authors contributed to the article and approved the submitted version.

## Funding

This work was supported by a NIH/NCI grant R01CA204856. YS received a CTSI A-PReP Fellowship. YH receives 3M Fellowship. RH also receives support from a research grant from the Avon Foundation for Women; a NIH/NCI grant R01CA229618; a University of Minnesota Faculty Research development grant and a University of Minnesota Grant-in-Aid award.

## Conflict of Interest

The authors declare that the research was conducted in the absence of any commercial or financial relationships that could be construed as a potential conflict of interest.

## References

[1] Wang F Liang R Soibam B Yang J Liu Y. Coregulatory Long Non-Coding RNA and Protein-Coding Genes in Serum Starved Cells. Biochim Biophys Acta - Gene Regul Mech (2019) 1862:824–95. 10.1016/j.bbagrm.2018.11.004 30503397

[2] Kopp F Mendell JT. Functional Classification and Experimental Dissection of Long Noncoding Rnas. Cell (2018) 172:393–407. 10.1016/j.cell.2018.01.011 29373828 PMC5978744

[3] Wang KC Chang HY. Molecular Mechanisms of Long Noncoding RNAs. Mol Cell (2011) 43:904–14. 10.1016/j.molcel.2011.08.018 PMC3199020 21925379

[4] Gutschner T Diederichs S. The Hallmarks of Cancer: A Long non-Coding RNA Point of View. RNA Biol (2012) 9:703–19. 10.4161/rna.20481 PMC3495743 22664915

[5] Mitra SA Mitra AP Triche TJ. A Central Role for Long Non-Coding RNA in Cancer. Front Genet (2012) 3:17. 10.3389/fgene.2012.00017 22363342 PMC3279698

[6] Huang Y Ling A Pareek S Huang RS. Oncogene or Tumor Suppressor? Long Noncoding RNAs Role in Patient’s Prognosis Varies Depending on Disease Type. Transl Res (2020) 230:98–110. 10.1016/j.trsl.2020.10.011 33152534 PMC7936950

[7] Schneider C King RM Philipson L. Genes Specifically Expressed At Growth Arrest of Mammalian Cells. Cell (1988) 54:787–93. 10.1016/S0092-8674(88)91065-3 3409319

[8] Ji J Dai X Yeung SCJ He X. The Role of Long Non-Coding RNA GAS5 in Cancers. Cancer Manag Res (2019) 11:2729–37. 10.2147/CMAR.S189052 PMC6497482 31114330

[9] Mourtada-Maarabouni M Pickard MR Hedge VL Farzaneh F Williams GT. GAS5, a Non-Protein-Coding RNA, Controls Apoptosis and is Downregulated in Breast Cancer. Oncogene (2009) 28:195–208. 10.1038/onc.2008.373 18836484

[10] Luo G Liu D Huang C Wang M Xiao X Zeng F. Lncrna GAS5 Inhibits Cellular Proliferation by Targeting P27kip1. Mol Cancer Res (2017) 15:789–99. 10.1158/1541-7786.MCR-16-0331 28396462

[11] Chen L Yang H Yi Z Jiang L Li Y Han Q. LncRNA GAS5 Regulates Redox Balance and Dysregulates the Cell Cycle and Apoptosis in Malignant Melanoma Cells. J Cancer Res Clin Oncol (2019) 145:637–52. 10.1007/s00432-018-2820-4 PMC6394673 30569211

[12] Pickard MR Mourtada-Maarabouni M Williams GT. Long Non-Coding RNA Gas5 Regulates Apoptosis in Prostate Cancer Cell Lines. Biochim Biophys Acta - Mol Basis Dis (2013) 1832:1613–23. 10.1016/j.bbadis.2013.05.005 23676682

[13] Chen Z Pan T Jiang D Jin L Geng Y Feng X. The Lncrna-GAS5/Mir-221-3p/DKK2 Axis Modulates Abcb1-Mediated Adriamycin Resistance of Breast Cancer Via the Wnt/β-Catenin Signaling Pathway. Mol Ther - Nucleic Acids (2020) 19:1434–48. 10.1016/j.omtn.2020.01.030 PMC7056627 32160712

[14] Gu J Wang Y Wang X Zhou D Shao C Zhou M. Downregulation of LncRNA Gas5 Confers Tamoxifen Resistance by Activating miR-222 in Breast Cancer. Cancer Lett (2018) 434:1–10. 10.1016/j.canlet.2018.06.039 29969658

[15] Nath A Lau EYT Lee AM Geeleher P Cho WCS Huang RS. Discovering Long Noncoding RNA Predictors of Anticancer Drug Sensitivity Beyond Protein-Coding Genes. Proc Natl Acad Sci USA (2019) 116:22020–9. 10.1073/pnas.1909998116 PMC6825320 31548386

[16] Siegel RL Miller KD Jemal A. Cancer Statistics, 2017. CA Cancer J Clin (2017) 67:7–30. 10.3322/caac.21387 28055103

[17] Kirby M Hirst C Crawford ED. Characterising the Castration-Resistant Prostate Cancer Population: A Systematic Review. Int J Clin Pract (2011) 65:1180–92. 10.1111/j.1742-1241.2011.02799.x 21995694

[18] National Comprehensive Cancer Network. Prostate Cancer (version 2.2021) (2021). Retrieved from https://www.nccn.org/professionals/physician_gls/pdf/prostate.pdf

[19] Varnai R Koskinen LM Mäntylä LE Szabo I Fitzgerald LM Sipeky C. Pharmacogenomic Biomarkers in Docetaxel Treatment of Prostate Cancer: From Discovery to Implementation. Genes (Basel) (2019) 10:599. 10.3390/genes10080599 PMC6723793 31398933

[20] Zhu Y Liu C Nadiminty N Lou W Tummala R Evans CP. Inhibition of Abcb1 Expression Overcomes Acquired Docetaxel Resistance in Prostate Cancer. Mol Cancer Ther (2013) 12:1829–36. 10.1158/1535-7163.MCT-13-0208 PMC3947549 23861346

[21] Lombard AP Liu C Armstrong CM Cucchiara V Gu X Lou W. Abcb1 Mediates Cabazitaxel–Docetaxel Cross-Resistance in Advanced Prostate Cancer. Mol Cancer Ther (2017) 16:2257–66. 10.1158/1535-7163.MCT-17-0179 PMC5628132 28698198

[22] Geeleher P Cox NJ Huang RS. Clinical Drug Response Can be Predicted Using Baseline Gene Expression Levels and In Vitro Drug Sensitivity in Cell Lines. Genome Biol (2014) 15:1–12. 10.1158/1538-7445.AM2014-5561 PMC4054092 24580837

[23] Nyquist MD Li Y Hwang TH Manlove LS Vessella RL Silverstein KAT. Talen-Engineered AR Gene Rearrangements Reveal Endocrine Uncoupling of Androgen Receptor in Prostate Cancer. Proc Natl Acad Sci USA (2013) 110:17492–7. 10.1073/pnas.1308587110 PMC3808622 24101480

[24] Lin HM Castillo L Mahon KL Chiam K Lee BY Nguyen Q. Circulating microRNAs are Associated With Docetaxel Chemotherapy Outcome in Castration-Resistant Prostate Cancer. Br J Cancer (2014) 110:2462–71. 10.1038/bjc.2014.181 PMC4021524 24714754

[25] Sharma NS Gnamlin P Durden B Gupta VK Kesh K Garrido VT. Long non-Coding RNA Gas5 Acts as Proliferation “Brakes” in CD133+ Cells Responsible for Tumor Recurrence. Oncogenesis (2019) 8:68. 10.1038/s41389-019-0177-4 31740660 PMC6861230

[26] Yacqub-Usman K Pickard MR Williams GT. Reciprocal Regulation of GAS5 LncRNA Levels and Mtor Inhibitor Action in Prostate Cancer Cells. Prostate (2015) 75:693–705. 10.1002/pros.22952 25650269

[27] Li W Zhai L Wang H Liu C Zhang J Chen W. Downregulation of LncRNA Gas5 Causes Trastuzumab Resistance in Breast Cancer. Oncotarget (2016) 7:27778–86. 10.18632/oncotarget.8413 PMC5053687 27034004

[28] Giatromanolaki A Fasoulaki V Kalamida D Mitrakas A Kakouratos C Lialiaris T. CYP17A1 and Androgen-Receptor Expression in Prostate Carcinoma Tissues and Cancer Cell Lines. Curr Urol (2019) 13:157–65. 10.1159/000499276 PMC6944932 31933595

[29] Chou CW Wang CC Wu CP Lin YJ Lee YC Cheng YW. Tumor Cycling Hypoxia Induces Chemoresistance in Glioblastoma Multiforme by Upregulating the Expression and Function of ABCB1. Neuro Oncol (2012) 14:1227–38. 10.1093/neuonc/nos195 PMC3452342 22946104

[30] Fultang N Illendula A Lin J Pandey MK Klase Z Peethambaran B. Ror1 Regulates Chemoresistance in Breast Cancer Via Modulation of Drug Efflux Pump Abcb1. Sci Rep (2020) 10:1821. 10.1038/s41598-020-58864-0 32020017 PMC7000766

[31] Vaidyanathan A Sawers L Gannon AL Chakravarty P Scott AL Bray SE. Abcb1 (Mdr1) Induction Defines a Common Resistance Mechanism in Paclitaxel- and Olaparib-Resistant Ovarian Cancer Cells. Br J Cancer (2016) 115:431–141. 10.1038/bjc.2016.203 PMC4985349 27415012

[32] Lin X Lin W Ku YS Wong FL Li MW Lam HM. Analysis of Soybean Long non-Coding RNAs Reveals a Subset of Small Peptide-Coding Transcripts1[Open]. Plant Physiol (2020) 182:1359–74. 10.1104/PP.19.01324 PMC7054870 31882456

