# Supplementary material for: A Long Noncoding RNA, GAS5 Can Be a Biomarker for Docetaxel Response in Castration Resistant Prostate Cancer
Source: Front Oncol. 2021 May 21;11:675215. doi: 10.3389/fonc.2021.675215 (PMC8176853; doi:10.3389/fonc.2021.675215)
Supplement: Supplementary file 1 [file DataSheet_1.docx]

**Supplemental Materials**


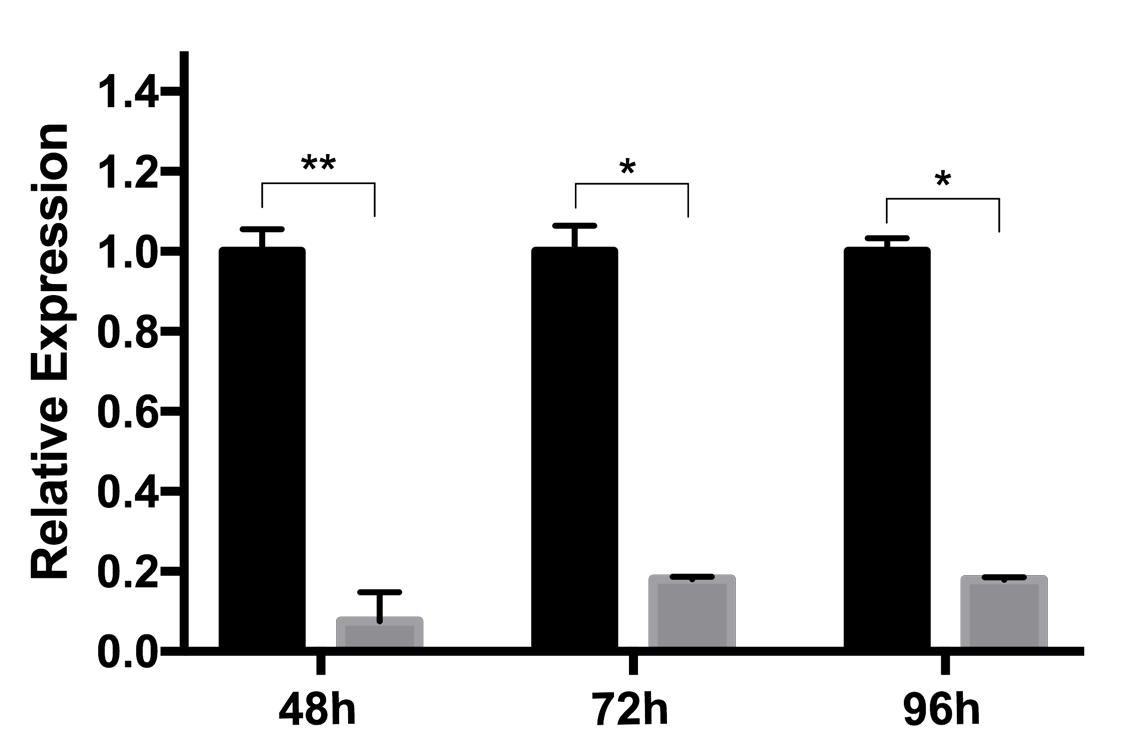


Fig. S1. qPCR results showing the relative expression of *GAS5* in R1D567 48h, 72h, and 96h after *GAS5* knockdown using siRNA pool. Black bars indicate *GAS5* expression levels in scramble control R1D567, while grey bars indicate the *GAS5* expression levels in *GAS5* knockdown R1D567. The expression level was normalized to *GAS5* expression in scramble control at each time point.

***P* < 0.01, ****P* < 0.001

(A)

(B)


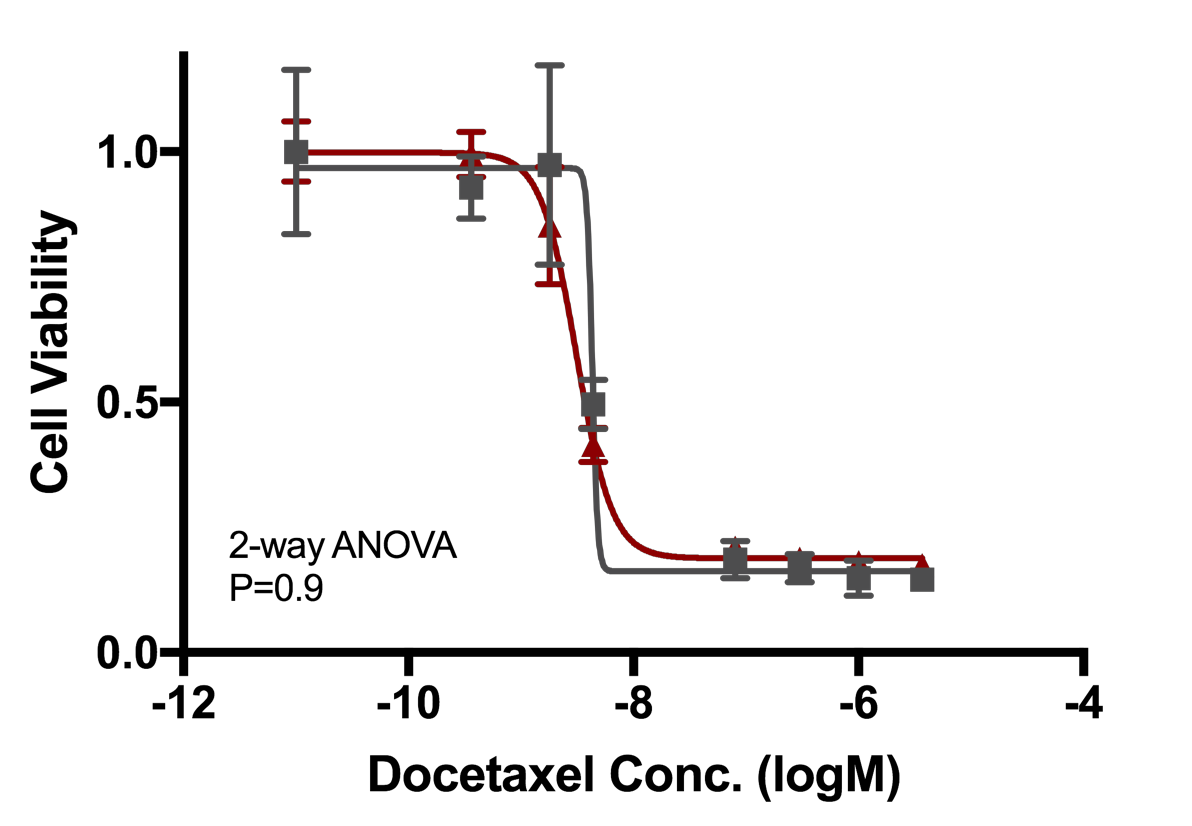

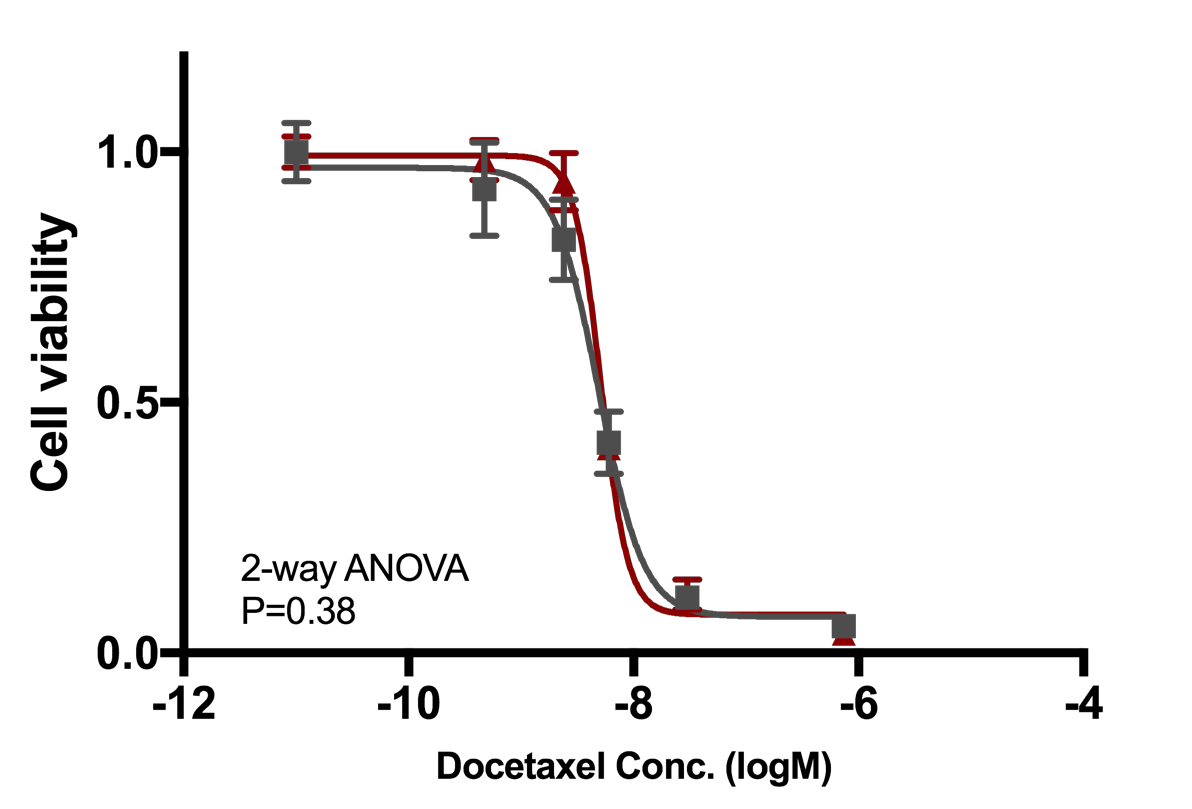


Fig. S2. Docetaxel dose response curves at 72 hours in (A) R1D567 and (B) DU145. Y-axis indicates relative cell viability normalized to cell viability in no drug treatment group. X-axis indicates different docetaxel concentrations that the cells were treated with. Red line represents the scramble control lines while blue line represents *GAS5* knockdown lines.

(A)


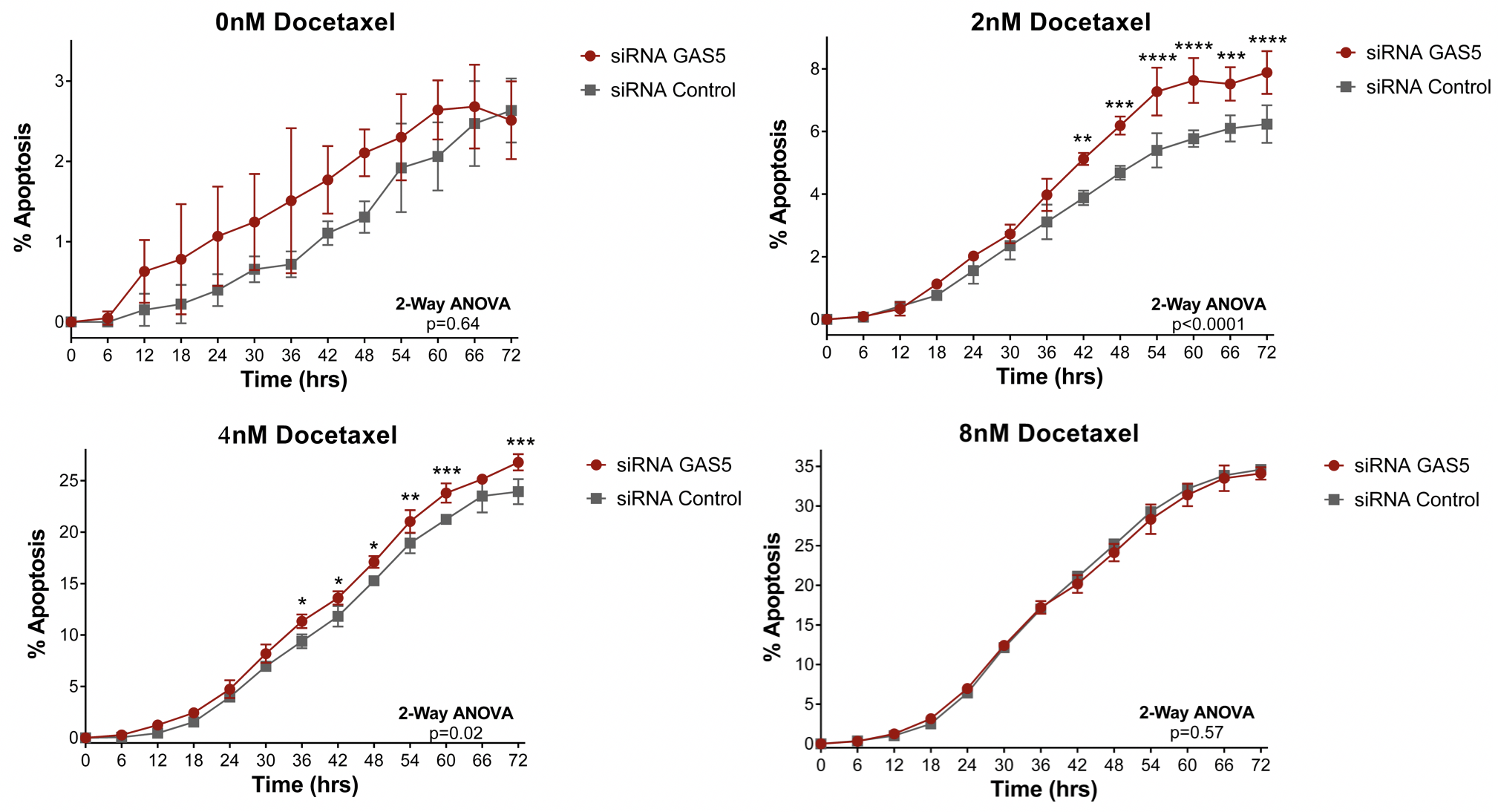


(B)


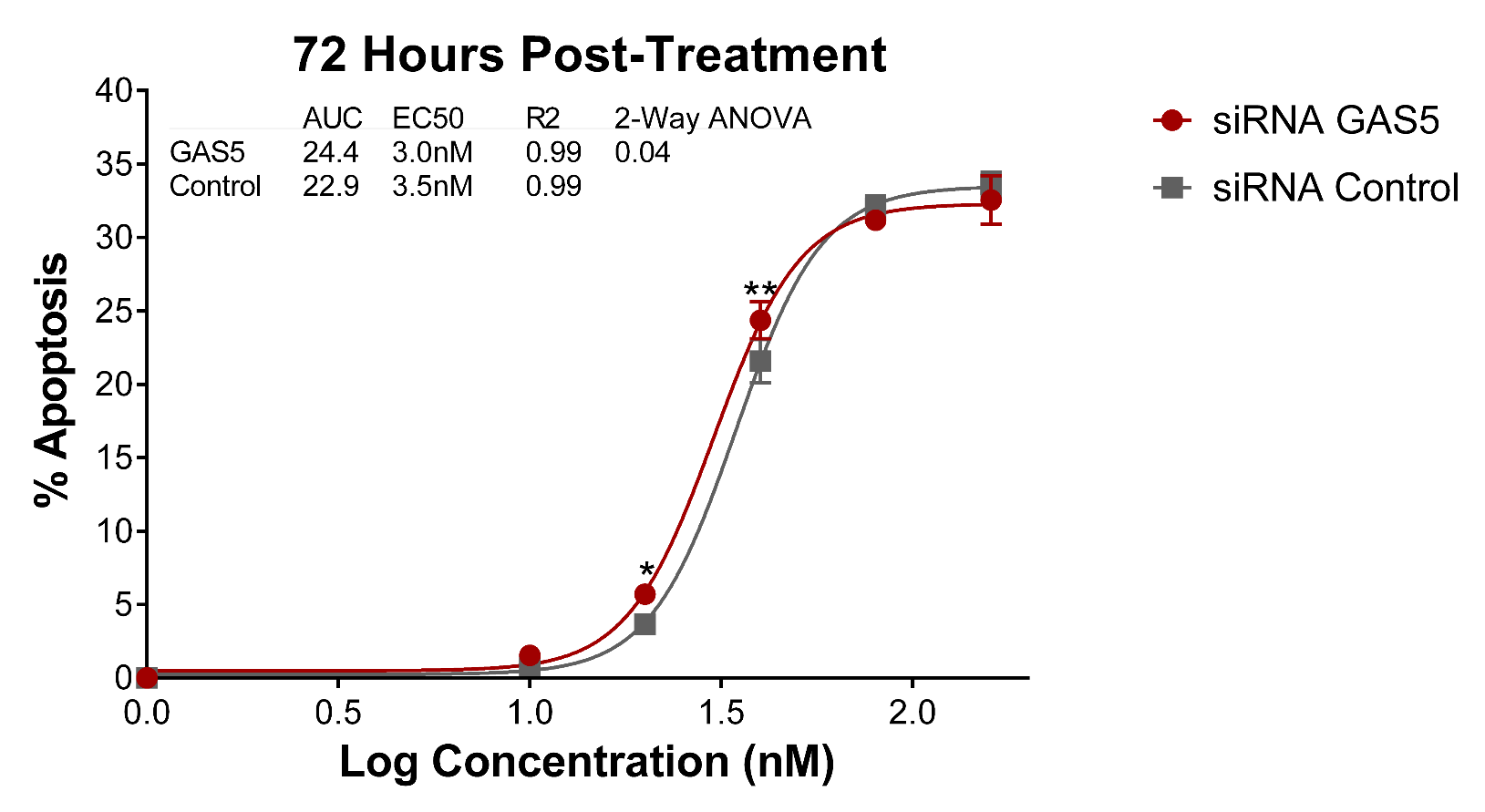


Fig. S3. Percentage apoptosis in siRNA *GAS5* group (red line) and siRNA control group (grey line) exposed to different concentrations of docetaxel. (A) % apoptosis across time in different docetaxel concentration groups. Y-axis indicates Percent apoptosis which was calculated by dividing the total number of GFP-positive cells by the total cell count and expressed as a percentage by multiplying by 100. X-axis indicates time post docetaxel treatment. (B) % apoptosis at 72 hours across different docetaxel concentrations. Y-axis indicates Percent apoptosis. X-axis indicates different concentrations of docetaxel that the cells were treated with. Results are reported as a mean and standard deviation of two independent biological experiments, each containing three technical replicates for each experimental condition.

**P* < 0.05, ***P* < 0.01


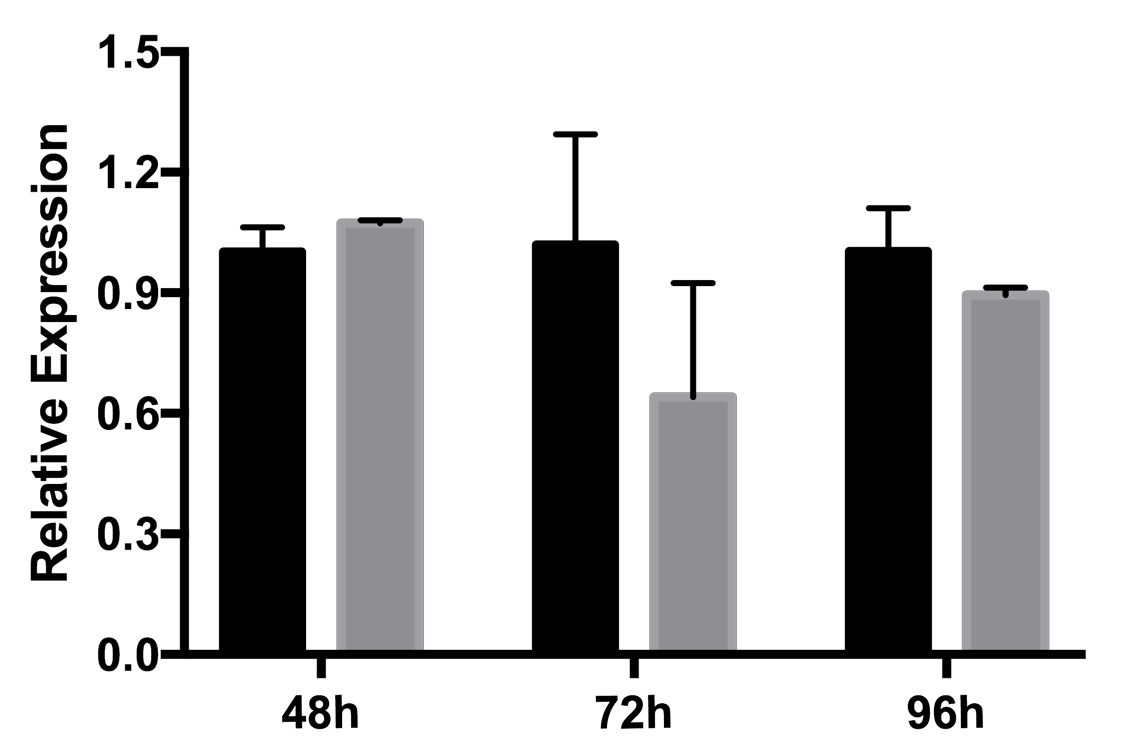


Fig. S4. qPCR results showing the relative expression of *ABCB1* in R1D567 48h, 72h, and 96h after *GAS5* knockdown using siRNA pool. Black bars indicate relative *ABCB1* expression levels in scramble control R1D567, while grey bars indicate the relative *ABCB1* expression levels in *GAS5* knockdown R1D567. The difference within at each time point is not significant.
